# Effect of in-hospital training in newborn resuscitation on the competence of health-care workers in resuscitating newborn infants at birth at Mboppi Baptist Hospital, Douala, Cameroon

**DOI:** 10.11604/pamj.2022.42.169.32816

**Published:** 2022-07-01

**Authors:** Hildegarde Nvonako, Ednah Ojee, Moses Masika, Arsène Sandie, Dalton Wamalwa, Aggrey Wasunna

**Affiliations:** 1 Nkwen Baptist Hospital, Bamenda, Cameroon,; 2 Department of Paediatrics and Child Health, University of Nairobi, Nairobi, Kenya,; 3 Kenya AIDS Vaccine Initiative (KAVI) Institute of Clinical Research, University of Nairobi, Nairobi, Kenya,; 4 African Population and Health Research Center, West Africa Regional Office, Dakar, Senegal

**Keywords:** Knowledge, skills, health care workers, newborn resuscitation, simulations

## Abstract

**Introduction:**

neonatal mortality accounts for the most significant proportion of under-five mortality worldwide, as in Cameroon. Birth asphyxia is the leading cause of neonatal deaths in Cameroon. Training of health care workers (HCWs) in newborn resuscitation reduces neonatal morbidity and mortality. In this study, we evaluated the effect of in-hospital training on the competence (knowledge and skills) of HCWs in newborn resuscitation at Mboppi Baptist Hospital, Douala, Cameroon.

**Methods:**

this was a quasi-experimental study done in five weeks, in which we compared knowledge and skills before and after training. Assessment of knowledge and skills of HCWs in newborn resuscitation was done before training (simulations) and a week after training using World Health Organization (WHO) adapted Emergency Triage Assessment and Treatment (ETAT+) standard tool. Three key informant interviews (KIIs) and a focused group discussion (FGD) were held to determine barriers to effective newborn resuscitation. Data were analyzed using R software version 3.6.2. McNemar test and Cohen´s Kappa were used to analyze quantitative data, while major themes from KIIs and FGDs were selected for qualitative data.

**Results:**

we enrolled 30 HCWs, each HCW was observed twice, a total of 60 deliveries observed before and 60 after training. Sixteen HCWs (53%) showed adequate knowledge before and after training. Median scores for skills significantly increased by 28% (p<0.00054) for real-life observations and 26% (p=0.0004) for newborn resuscitation scenario simulations. The main barriers to adequate newborn resuscitation were inadequate knowledge, equipment, shortage of trained staff and poor teamwork between midwives and anesthetists.

**Conclusion:**

in-hospital training on newborn resuscitation improved the skills of HCWs but had no significant effect on their knowledge on newborn resuscitation. We would recommend that in-hospital training in newborn resuscitation be done often for HCWs.

## Introduction

Globally, under-five mortality has decreased from 1990 to 2018, and so has neonatal mortality though at a slower rate [1]. Despite strategies and interventions to reduce neonatal mortality, the latter still accounts for the most significant burden of under-five mortality [1]. Neonatal mortality makes up to 40% of under-five deaths by 1990 and 47% of the same by 2018 [1]. The World Health Organization (WHO) estimates that each year, about 3 million neonates die within their first month of life and that 98% of these deaths occur in developing countries, especially in Africa and Asia [2]. Each year about 2 million babies die during labour, childbirth, or on the first day of life (1.3 million intrapartum still births; 1 million newborn deaths in the first 24 hours) [2]. Another 1 million die before reaching the first week of life [2]. Newer estimates show a global mortality rate of 18 per 1000 live births [3]. Globally an estimated 2.5 million newborns died in their first month of life in 2018 - approximately 7000 deaths per day [3].

According to the Cameroon demographic health surveys, under-five and neonatal mortalities in 2018 were 79/1000 and 32/1000 respectively. Cameroon is therefore among the 53 countries in which progress needs to be accelerated to reach the sustainable development goal target of 25 or fewer deaths per 1000 live births in the under-five population by 2030 [1]. Almost half of these under-five deaths will be newborns whose deaths can be prevented by reaching high coverage of quality antenatal care, skilled care at birth, postnatal care for mother and baby, and care of small and sick newborns [1].

Andreas *et al*. in an urban health facility in Cameroon, found the prevalence of birth asphyxia to be 80.5/1000 live births, with hospital mortality of 6.7% and morbidity (neurologic defects) of 12.2% [4]. The global burden of birth asphyxia has led to various studies being carried out with the aim of reducing neonatal morbidity and mortality due to birth asphyxia. A systematic review on neonatal resuscitation training in health care facilities reports that training of birth attendants could avert 30% of intrapartum-related neonatal deaths [5].

Birth asphyxia is as a result of impaired placental or pulmonary gas exchange. It can lead to the decrease of oxygen (hypoxia) or excess carbon dioxide levels (hypercarbia) in the blood [6]. Hypoxia can diminish myocardial function resulting in hypotension and ischemia [6]. Ischaemia can cause further compromise and disrupt the supply of nutrients and removal of metabolic products (lactic acid and carbon dioxide) due to impaired oxygen delivery [6]. Adverse birth outcomes are the most significant drain on human capital due to death and disability [1]. Birth asphyxia is the third leading cause of neonatal mortality worldwide and the first leading cause of neonatal deaths in Cameroon, yet it can be prevented through good obstetric care and its consequences attenuated by effective and timely basic newborn resuscitation, a low-cost intervention [2].

Several nations have adopted the WHO guidelines for newborn resuscitation including Cameroon, in June, 2017. However, the challenge in Cameroon remains the translation of these guidelines into a well-structured and practical approach that can easily be used by health personnel in resuscitating an asphyxiated newborn. The Baptist Hospitals in Cameroon, in an attempt to bridge this gap, introduced the Advanced Life Support in Obstetrics (ALSO) guideline for resuscitation which focuses on obstetrics rather than neonatal emergencies. In ALSO training, neonatal resuscitation skills, unlike obstetrics skills, are not assessed at the end of the course to ascertain immediate understanding and retention.

In 2018, Mboppi Baptist Hospital Douala (MBHD), Cameroon recorded a total of 3,456 deliveries (both vaginal and caesarian) amongst which 332 babies (10% ) were born asphyxiated (Apgar less than seven at first minute) and 72 (2%) neonatal deaths recorded (which included still-births, inevitable abortions and intra uterine foetal demise). Competence as defined by the English Oxford dictionary is 'having the necessary ability, knowledge or skill to do something successfully; an acceptable and satisfactory though not outstanding ability´. Studies have been carried out to assess competence of HCWs in relation to knowledge and skills in newborn resuscitation [4-8]. Assessment of knowledge in these studies ranged from semi-structured to multiple choice standardized questions while assessment of skills was done using WHO simulation tools for newborn resuscitation. In our study, competence was defined as having both knowledge and skills in newborn resuscitation. Given that we did not come across a composite tool in our literature search that assesses both knowledge and skills, we assessed knowledge based on the pass level of the neonatal resuscitation training programme validated tool and skills based on the pass criteria of WHO adapted ETAT+ tool (Annex 1).

Training health workers in neonatal resuscitation using simulations is one of the interventions that has proven to be directly linked to improving neonatal outcomes [3]. This study was designed to assess the effect of in-hospital training (using simulations) in newborn resuscitation on the competence (knowledge and skills) of health workers in resuscitating newborns in maternity and theatre of MBHD, Cameroon.

## Methods

**Study design and period:** this was a quasi-experimental study comparing level of knowledge and skills before and after training. Both quantitative and qualitative methods of data collection were used. The study was carried out from November to December 2019 for five weeks.

**Study population:** the study population was HCWs (midwives, nurses and paramedical staff) in labour wards at MBHD, Cameroon. The paramedical staff in this hospital are HCWs with minimal training and responsible for taking patients´ vital signs. We included all HCWs who had worked in the labour wards for at least four weeks and who gave consent. We excluded HCWs who were part-time workers and those absent (on leave) during the study period.

**Study location:** the Cameroon Baptist Convention Health Services provides healthcare in six of the ten regions in Cameroon. It comprises five hospitals, 26 secondary health centers staffed by nurses, and more than 50 primary health centers. MBHD is the second largest one of these five hospitals. Mboppi Baptist Hospital is a fast-growing hospital and a tertiary referral centre in the Cameroonian cosmopolitan economic capital of Douala, the second largest city in Cameroon with about 1.3 million inhabitants. MBHD has a bed capacity of 134 and more than 1000 patients daily. MBHD has about 500 staff, including 15 medical officers, nine consultants (2 surgeons, 2 gynaecologists, 2 internists, 1 cardiologist, 1 dentist and 1 paediatrician). The maternity department has two obstetrics and gynaecology consultants, 33 nurses and midwives and a monthly average of 275 deliveries.

**Sample size determination:** formula for pre and post comparison studies was used to calculate sample size. Our study had two primary outcomes; knowledge and skills. To assess for knowledge, we planned to assess all HCWs who met the inclusion criteria. Knowing that the number of HCWs in the facility ranged between 30-35, this same number was to be assessed before and after training for knowledge. Hence the sample size calculation was to determine the number of newborn deliveries to be observed as conducted by these HCWs before and after the training. Using skill score findings from previous before and after studies to calculate sample size, we got sample sizes in the 40´s, which we found insufficient to observe all HCWs. However, using the scores on knowledge pre (75%) and post (95%) training to calculate sample size based on results obtained by Singhal *et al*. Kenya [8], we obtained a satisfactory sample size as shown below, which could help us observe all HCWs at least twice both before and after the training.

The confidence interval used was 95% with power of 90%.


$$n=\frac{{\left\{\sqrt[{u}]{\pi_{1}(1-\pi_{1})+\pi_{0}(1-\pi_{1})}+\sqrt[{\nu }]{2\pi (1-\pi )}\right\}}^{2}}{{\left(\pi_{0}-\pi_{1}\right)}^{2}}$$

Source: Kirkwood and Sterne medical statistics. Where: u = power 90%; v = 1.96 (95% C.I); π_0_= level of knowledge on newborn resuscitation before training (0.75); π_1_= level of knowledge on newborn resuscitation after training (0.95).


$$n=\frac{\left(\pi_{0}+\pi_{1}\right)}{2}=60\;[newborn\text{ resuscitations for each phase(before and after training)}]$$

Therefore, each of the health workers was observed doing a resuscitation 2 times both before and after the training. The aim was to observe all 30 HCWs who work in the labour wards. Observing each of them once will give us 30 observations, so to meet up with the sample size of 60, each was observed twice.

**Figure 1:**
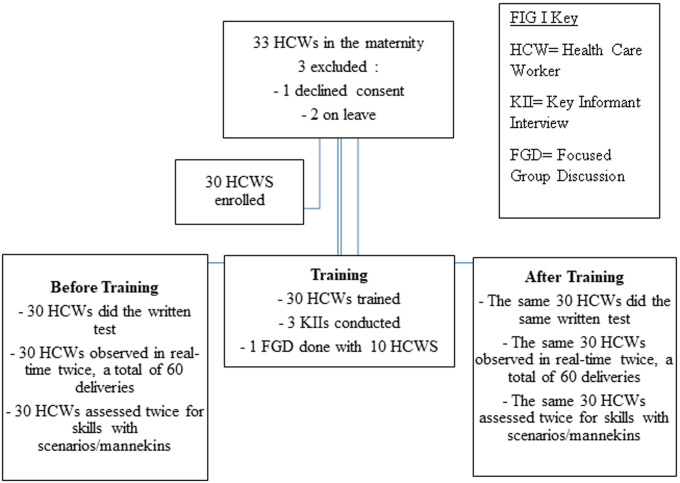
participants’ enrollment flow diagram

**Recruitment procedure:** thirty HCWs were enrolled into the study and each HCW was observed doing a resuscitation two times before and two times after the training, a total of 120 observations (Figure 1). The observations were carried out by the principal investigator (PI), a resident in the paediatrics and child health programme at the University of Nairobi, a certified trainer of the advanced life support obstetric course in Cameroon and Kenya, as well as an instructor of the Emergency Triage Assessment and Treatment (ETAT+) Course in Kenya. Each participant was observed either in the labour ward or maternity theatre while conducting newborn resuscitation. The PI was in the rota of the maternity staff such that she rotated in all shifts alternately and could observe all HCWs during their different shifts either mornings, afternoons or night shifts as well as over the weekends to minimize Hawthorn effect (the tendency of an individual to improve his/her performance once he/she knows they are being observed). Each observation going through 10 minutes. On average, each morning shift had 4 HCWs in the delivery room, while afternoon and night shifts had 2 workers each in the delivery room, with an average of 8 deliveries daily.

**Study procedure:** the study procedure involved 3 phases:

**Pre-intervention phase (2 weeks duration):** for knowledge assessment, a standardised questionnaire from Neonatal Resuscitation in Paediatrics (NRP) textbook was administered to the 30 participants to assess baseline knowledge on newborn resuscitation. A score of 70% or higher was considered adequate knowledge (standard pass mark set by NRP tool). For skills assessment, the PI observed 30 participants perform resuscitation as part of their routine duties in labour wards and theatres over a period of two weeks. Each observation was checked against the WHO ETAT+ checklist (Annex 1). The WHO ETAT+ training package has 5 key steps for newborn resuscitation: drying the baby, assessing the airway, assessing breathing, giving 30 ventilation breaths in a minute and assessing circulation (Annex 1). Those who achieved all 5 key steps in newborn resuscitation as per the WHO ETAT+ checklist were considered as having adequate skills. For each participant the pre-training skill score was the mean of the scores obtained in the two observations. During the same two week period, the PI conducted one-on-one assessment of skills of the 30 HCWs using simulation scenarios (two scenarios per HCW, one of a baby born without meconium and the other of a baby born with meconium) as per the ETAT+ checklist. The scores in each scenario for each participant were obtained.

**Intervention phase (3 days within 1 week):** the 30 HCWs were divided into 3 groups of 10 participants. The PI trained each group in a day within a period of one week using the WHO ETAT+ guideline on newborn resuscitation. Each training session lasted 1.5 hours/day and comprised of: a 35 minutes didactic lecture using WHO ETAT+ MS Powerpoint presentation on newborn resuscitation; ten minutes of skills demonstration with mannequins using 2 case scenarios (meconium and no meconium) from WHO ETAT+ MS Powerpoint slides; fourty-five minutes for practice by all participants (4.5 minutes each).

**Post-intervention phase (within 1 week after the intervention and for a duration of 2 weeks):** the PI conducted a post intervention re-assessment of knowledge on newborn resuscitation using the same standardized questionnaire used in the baseline assessment. A score equal of 70% or more was considered adequate knowledge. All participants were re-observed while conducting newborn deliveries and their skills on newborn resuscitation checked against the WHO ETAT+ checklist. The PI observed each HCW attending to a newborn baby on two separate occasions. One-on-one skills assessment of HCWs with mannequins was also done for each of the 30 HCWs using the two newborn resuscitation scenarios as taught in the training - newborn with and without meconium. The skills assessment and scoring in this phase was done exactly as in phase one.

Three key informant interviews (KIIs) with the senior nurse supervisor, nurses in charge of labour ward and theatre were conducted. This was to identify the barriers to effective newborn resuscitation encountered in their practice and unit, using the questionnaire. The interviews were recorded on a notebook. We also held a focused group discussion (FGD) with a group of 10 participants on barriers they face in resuscitating newborns. From the three groups of 10 participants/group we trained, we randomly selected one group. The session took approximately 20 minutes and was held in a conference room at the hospital. A discussion guide was used and norms of the focus group discussion were agreed upon by the participants at the beginning. After obtaining consent, the FGD was recorded with a voice recorder. A major theme emerging approach was used in the analysis of this focus group discussion.

Trustworthiness of the qualitative aspect of the study was achieved by the following three methods: prolonged engagement - the PI spending extended time with participants in their natural environment and everyday world in order to gain a better understanding of behaviour, values and social relationships in a social context. This helps in drawing out true observations and conclusion; balance - the study used a mixed methods design (both qualitative and quantitative aspects) to achieve impartiality. This improves validity, reliability and authenticity of qualitative research; corroboration - the PI in the interviews and FGDs avoided leading questions and questions with double meanings. By involving many respondents this helped the PI to corroborate information amongst the sources hence proving the information true or false.

**Ethical considerations:** we obtained approvals from the Kenyatta National Hospital - University of Nairobi Ethics and Research Committee (UON/CHS/PAED/5/1), from the Cameroon Baptist Convention Health Services, Institutional Review Board (IRB2019-28), as well as from the administrator of MBHD where the study was conducted. Informed consent was sought from the HCWs as a group in a meeting. Objectives of the study, risks, safety and benefits were explained and group verbal consent was obtained. They were assured that participation was voluntary and that refusal to take part in the study would not in any way compromise their position at work. All personal information of HCWs was kept strictly confidential. The principal investigator offered assistance in newborn care to midwives when the need arose and when called upon for help, keeping a check on the newborn´s safety during resuscitation.

**Data management and analysis:** data entry was done using the CSPro software version 7.3.1 and all data analysis was done using the R software version 3.6.2. Frequencies and proportions were used to summarize categorical data. Median and inter-quartile range (IQR) were used to describe numerical variables. The non-parametric Wilcoxon signed rank test was used to compare paired (before and after) continuous data. The McNemar test was used to compare the paired scores (before and after). Cohen´s Kappa coefficient was used to measure the agreement between scores pre and post training in order to determine the true ratings for each participant and to tell if the ratings before and after training were truly similar or truly different. The answers from KIIs and FGDs were written down and recorded respectively by the PI. Thereafter, the answers with the highest frequency of occurrence which arose from these were highlighted. These answers were then documented using selected quotes to represent each theme.

## Results

Thirty HCWs participated in this study (Table 1). Each was observed attending to newborns twice before and twice after training.

**Table 1:** baseline characteristics of health workers assessed for knowledge and skills in newborn resuscitation before and after training at Mboppi Baptist Hospital, Douala, Cameroon (N=30)

Characteristics	n (%)
**Gender**	
Male	6 (20)
Female	24 (80)
**Cadre**	
Nurse midwife	16 (53)
Registered nurse	3 (10)
Paramedical staff	11 (37)
**Education**	
Primary	5 (17)
Secondary	13 (43)
Tertiary	12 (40)
Previous training in newborn resuscitation	17 (57)
**Level of confidence in newborn resuscitation**	
Very confident	10 (33)
Somewhat confident	17 (57)
Not confident	3 (10)

**Knowledge assessment of health care workers:** in the overall knowledge assessment, the same number of health workers, 16 (53%) got a pass mark in the written assessment before and after the training. The McNemar´s test (p-value =1.00) for paired proportion indicated no significant difference in the proportion of pass marks before and after training. Moreover, Cohen´s Kappa agreement was 0.33, indicating a fair agreement between the score before and after training. These all indicate that the training did not have a significant effect on the overall knowledge of health workers on newborn resuscitation.

**Skill assessment of health care workers:** the median skill score of participants based on their real-time practice of newborn resuscitation was 54% (IQR=14) before the training and this increased to 82% (IQR=30) after the training. The Wilcoxon signed rank test was significant (p<0.0001), indicating a significant increase in skills acquired translated into real-life newborn resuscitation practice. Cohen´s Kappa inter-rater test coefficient for these values before and after was very small and close to zero (0.0034) showing that the values obtained before and after the training were genuinely different. In simulation assessment of the newborn resuscitation skills using scenario 1 (baby born without meconium), the median score for participants was 43% (IQR=28) before the training. This increased to 57% (IQR=28) after the training (p=0.008). For scenario 2 (baby born with meconium), the median skill score was 38% (IQR=25) before the training but increased to 75% (IQR=25) after the training (p<0.0001).

**Figure 2:**
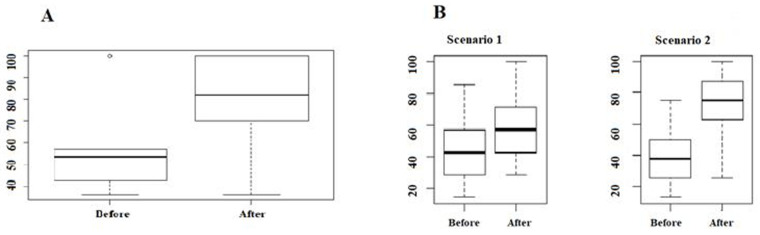
newborn resuscitation skill scores of health workers before and after training at Mboppi Baptist Hospital Douala, Cameroon (N=30)

The Wilcoxon signed rank test indicates that for both scenarios, the skill score significantly increased after training. Moreover, the Cohen´s Kappa inter-rater agreement for both scenarios was very small and close to zero (0.016 for scenario 1 and 0.084 for scenario 2), suggesting no agreement between the skill score outcome before and after the training. Meaning there is an actual difference between the scores before and after training. All these results are concordant and indicate that the training had a significant effect on the skills of HCWs (Figure 2).

**Qualitative analysis results:** the following themes were derived from 3 key informant interviews (head nurse of maternity; head nurse of theatre and senior nurse supervisor) on the challenges they face in-effective newborn resuscitation: inadequate equipment such as appropriate bags and masks for ventilation, insufficient knowledge on newborn resuscitation in health workers and low level of training of some staff in maternity. Proposed solutions to these challenges were providing appropriate equipment by hospital, frequent training and refresher courses on newborn resuscitation for maternity staff, and revising intake criteria by hospital management of staff who are to work in maternity. One key informant said: *'we need newborn resuscitation trainings more frequently in this hospital because the work is alot but trained midwives are few´*.

In the FGD, nurses and midwives expressed the following challenges faced in effectively resuscitating newborns: inadequate knowledge on newborn resuscitation in HCWs, inadequate equipments in theatre, inadequate training on newborn resuscitation and inadequate teamwork between theatre staff and midwives. One midwife in the FGD said: *'at times we feel we are not useful in theatre when we go to receive babies, because when a baby is born in theatre and does not cry, the anaesthetist will just push us away and take over as though we know nothing'*. Proposed solutions included: in-hospital drills with mannequins, training of midwives and anaesthetists on newborn resuscitation.

## Discussion

Our study sought to determine the effect of in-hospital training in newborn resuscitation on the competence of health workers in resuscitating newborns at MBHD, Cameroon. We assessed and compared the HCWs´ level of knowledge and skills before and after in-hospital training in newborn resuscitation. We also explored the barriers and solutions to effective newborn resuscitation in MBHD. The training improved HCWs skills but had no effect on their knowledge in newborn resuscitation. About half of the HCWs in this study attained a pass mark in the written test before and after the training. This demonstrated no significant effect on the overall level of knowledge in newborn resuscitation of HCWs after the training. These results were different from those obtained by Taksande *et al*. [9] in a study looking at the efficiency of the neonatal resuscitation training programme in a rural hospital in India where the pass rate almost doubled after the training. This difference could be because the education level of participants in both studies was not similar - the participants in our study were primarily nurses and midwives with highest level of education being a bachelor´s degree in nursing. Meanwhile, those in the study done in India were primarily postgraduate and final year undergraduate medical students. Secondly, the period between training and administration of the tests was also different. In our study, participants got the pre-test two weeks before the training and the post-test within one week after completing the training meanwhile, in the study by Taksande *et al*. participants got pre-test on the day of the training and the post-test immediately after the training, this could have favoured better scores in their post-test.

In assessing health care providers´ knowledge on newborn resuscitation in Kenya, a 10 multiple choice question standard test was given to them and those who scored >85 were considered successful. It was found that only 68 participants out of 192 (35.4%) had adequate knowledge on newborn resuscitation [10]. These included medical, clinical and nursing officers randomly selected from the 47 counties of Kenya. In Ghana, basic neonatal care module was taught as part of the American neonatal resuscitation training package and cognitive knowledge was evaluated by written pre and post training tests. All 271 professionals enrolled (midwives, physicians, nurses and nurse anaesthetists) who went through the training had significant improvement (p<0.001) in median post training written test performance [7].

The participants´ skill scores in the real-time practice of newborn resuscitation significantly increased by a quarter after the training, These results were similar to those obtained by Opiyo *et al*. [11] in 2007, a study looking at the effect of a one-day newborn resuscitation training on health care workers at a maternity hospital in Kenya. They found a 66% improvement in the primary recommended steps in newborn resuscitation in the trained health workers. Looking at each of the primary resuscitation steps and how HCWs performed them before and after the training, we noted that HCWs performed best in 'suctioning a baby born in meconium before stimulation' and other steps - correct drying, stimulation, warmth provision and assessing breathing were also adequately achieved both before and after the training. Hence expectations could be that these primary resuscitation steps acquired by our HCWs will go a long a way to save newborns who will need such assistance. This was similar to results obtained by Msemo *et al*. in 2013 in Tanzania [12], who found a 7% increase in skills scored in proper suctioning and 41% improvement in skills scored in proper stimulation, after training birth attendants in a workshop named 'helping babies breathe'.

In simulation assessment of the newborn resuscitation skills using scenario 1 (baby born without meconium), participants´ skill scores significantly improved by 28% after the training. For scenario 2 (baby born with meconium), participants´ skill scores increased significantly by two thirds. This positive impact can still be compared to that obtained by Opiyo *et al*. looking at the critical steps achieved by HCWs in scenario 1 before, and after the training, we noted they performed best in 'giving ventilations with a bag and mask' but did poorly in circulation assessment. For scenario 2, HCWs performed well in 'suctioning what you can see' while the poorest skills noted were still 'assessing for circulation' both before and after the training. The general observation was that none of the HCWs achieved all critical steps in either scenarios before and after training. Moreover, the level of improvement in all but one of the critical steps after the training was satisfactory, hence an anticipated better neonatal outcome for babies who fail to breathe at birth. In Rwanda, 118 health trainees completed the helping the babies breathe course and pre and post training evaluations showed an increase in percentages of correct answers on written test from 77% to 91% (p<0.01) after training and a mean score of 89% on post evaluation skills; 64% of trainees achieving passing scores [13].

Monebenimp *et al*. suggested an urgent need to reinforce resuscitation skills of health workers of a level 1 facility in Cameroon, after a cross section observational study involving 10 health workers and 21 newborn resuscitation cases. They used the WHO Essential Newborn Care (ENC) checklist for skills observation. The study showed that the workers scored only 6 points out of 17, performing only 24% of expected tasks [14]. Circulation assessment, which remained unachieved by the majority of the participants, may be due to several reasons. Firstly, it maybe because the HCWs have not had much exposure to babies failing to breathe at birth to the extent of such needing cardiopulmonary resuscitation (in our study, 87% of babies cried immediately at birth and 73% of HCWs reported having conducted less than five resuscitations within the three months preceding the study). Secondly, the PI might not have explained circulation assessment very well or may not have emphasized it sufficiently during training.

These results showing a positive impact of the training on the skills of HCWs are similar to those obtained by Singhal *et al*. in 2012 in Kenya [8], where an improvement in the HCWs skill of ventilating with bag and mask was noted to have risen by 15% after training. In an unpublished study, Okara *et al*. in 2015 in Kenya also noted a 32% improvement in the use of bag valve masks in a study evaluating the effectiveness of in-hospital training in newborn resuscitation at Kisii Teaching and Referral Hospital. This step is acknowledged as the most critical and complex step in basic newborn resuscitation [6], hence improvement in skill through training is likely to lead to better neonatal outcomes.

The qualitative section of our study enabled us to engage the HCWs and some key informants in the hospital on what they perceived as barriers to adequate newborn resuscitation in their setting. The four major themes that arose as barriers from the FGD and KIIs were: lack of appropriate newborn resuscitation equipment, inadequate knowledge in newborn resuscitation, shortage of trained staff and insufficient midwife/anaesthetist teamwork in theatre. These barriers were similar to those found by Otido *et al*. in an unpublished study in 2010 in Garissa Level 5 Hospital in Kenya while assessing reasons for non-adherence to newborn resuscitation guidelines by HCWs.

During the FGD and KIIs we also engaged the participants to suggest solutions to the barriers they mentioned. Proposed solutions included: supply of appropriate equipment, refresher trainings and in-hospital drills on newborn resuscitation, recruitment of qualified staff (trained midwives) in maternity and joint trainings on newborn resuscitation for midwives and anaesthetists to enhance teamwork in resuscitating newborns in theatre.

**Study limitations:** the Hawthorne effect was anticipated because of the nature of the study. To minimize this, a gross explanation of the study without giving details of the steps that were being observed was given to participants. Also, the PI whom the participants were familiar with, joined the staff to work in the various shifts so that the staff would be free to perform their duties as usual. Hence the staff did not know exactly when they were being observed (during real life resuscitation). Moreover, the PI, the only trained personnel in ETAT+ in the facility, was the sole observer in this study. To minimize observer bias, each observation was done twice, and the average taken. Also, the analysis of the observations used the observer intra-reliability component of Cohen Kappa test to minimize the bias of a sole observer.

## Conclusion

In-hospital training on newborn resuscitation had a positive effect on the skills of health care workers in resuscitating newborns. The training however, did not have an effect on the knowledge of these HCWs in newborn resuscitation. Based on the identified barriers to effective newborn resuscitation, we would recommend a supply of appropriate resuscitation equipment such as appropriate neonatal sizes of bags and masks in the hospital´s delivery room and theatre. Also, refresher courses and in-hospital drills to be organized on newborn resuscitation for midwives and anaesthetists.

**Funding:** the data collection process was partially funded by the Pan African Medical Journal - Center for Publish Health Research and Information (Kenya).

### What is known about this topic

- Birth asphyxia accounts for the leading cause of neonatal deaths in Cameroon and third leading cause of the same worldwide;
- Training health care workers to promptly intervene with safe and effective skills in resuscitating asphyxiated newborns has been shown to reduce morbidity and mortality both in developed and developing settings.

### What this study adds

- Our study brought a well-structured and practical approach (WHO ETAT+ standard tool) in resuscitating newborns which did not exist in the study setting before. Participants´ performance showed that this approach was easily understood and used in effectively resuscitating newborns both in real-time practice and in simulation scenarios with mannequins. This suggests that these skills gained will go a long way in improving neonatal outcomes;
- Our study went beyond the traditional method of delivering ETAT+ training that is, mainly classroom and evaluation based solely on simulations with mannequins. In our study, we opted for in-hospital training and added real-time observation of participants conducting the skills on duty both before and after the training. This showed how the acquired skills were adequately translated into real time practice;
- Our study had a qualitative aspect which helped us understand possible barriers to effective newborn resuscitation in the facility and proposed solutions to these ones.

## Annex

**Annex 1**: WHO adapted ETAT+ Newborn Resuscitation Checklist for skills (real-time practice, NB: The five bold steps are mandatory/compulsory and achieving all five qualifies a HCW competent in newborn resuscitation skills) (PDF-82Kb)
